# Antithrombotic Strategies After Complex Percutaneous Coronary Intervention

**DOI:** 10.3390/jcm15135196

**Published:** 2026-07-02

**Authors:** Yasushi Ueki, Koichiro Kuwahara

**Affiliations:** Department of Cardiovascular Medicine, Shinshu University Hospital, Nagano 390-8621, Japan; kkuwah@shinshu-u.ac.jp

**Keywords:** percutaneous coronary intervention, antiplatelet therapy, anticoagulation, bleeding

## Abstract

Complex percutaneous coronary intervention (PCI) represents a growing proportion of contemporary coronary revascularization, driven by aging populations, increasing comorbidity burden, and advances in interventional techniques. Complex PCI encompasses a spectrum of anatomically and procedurally challenging lesions, including left main disease, bifurcation lesions requiring two-stent strategies, chronic total occlusions, long stent lengths, severe calcification requiring atherectomy, and multivessel revascularization. Antithrombotic therapy, comprising antiplatelet and anticoagulant agents, is essential for preventing stent thrombosis and other ischemic events in both the early and long-term phases after PCI. While antithrombotic therapy mitigates ischemic risks associated with complex PCI, these patients frequently carry a high bleeding risk, thus making the choice of antithrombotic regimen challenging. Recent guideline recommendations emphasize balancing ischemic and bleeding risks rather than relying solely on procedural complexity. This review synthesizes contemporary evidence, guideline recommendations, and clinical considerations for antithrombotic therapy after complex PCI.

## 1. Introduction

Advances in percutaneous coronary intervention (PCI), including refinements in stent technology, the adoption of intravascular imaging, and more effective adjunctive pharmacotherapy, have enabled operators to treat increasingly complex coronary lesions with high procedural success. Complex PCI constitutes roughly 30–40% of all PCI procedures and is now performed routinely in daily clinical practice [1,2]. Complex PCI includes a broad range of challenging scenarios such as multivessel disease, left main (LM) disease, bifurcation lesions, chronic total occlusion (CTO), and heavily calcified segments requiring atherectomy. These procedures are often associated with increased ischemic risks, including repeat revascularization, myocardial infarction (MI), and stent thrombosis. Historically, the perceived ischemic risk associated with complex PCI led to prolonged duration of dual antiplatelet therapy (DAPT). However, with the improved safety profile of new-generation drug-eluting stents and the growing recognition of the prognostic impact of bleeding events, current international guidelines recommend determining DAPT regimens based not only on ischemic risk but also on bleeding risk. This review provides a comprehensive overview of antithrombotic strategies after complex PCI, integrating evidence from major trials and guideline recommendations to inform clinical practice.

## 2. Review Methods

Our literature search was conducted primarily in PubMed using the terms “complex” or “complexity”, “percutaneous coronary intervention” or “PCI” or “revascularization”, and “antiplatelet treatment” or “antiplatelet therapy”. We focused on publications from 2010 to 2026. Study selection prioritized authoritative clinical guidelines, pivotal clinical trials, and high-quality observational studies relevant to the objective of this review.

## 3. Definition of Complex PCI

**Figure 1:**
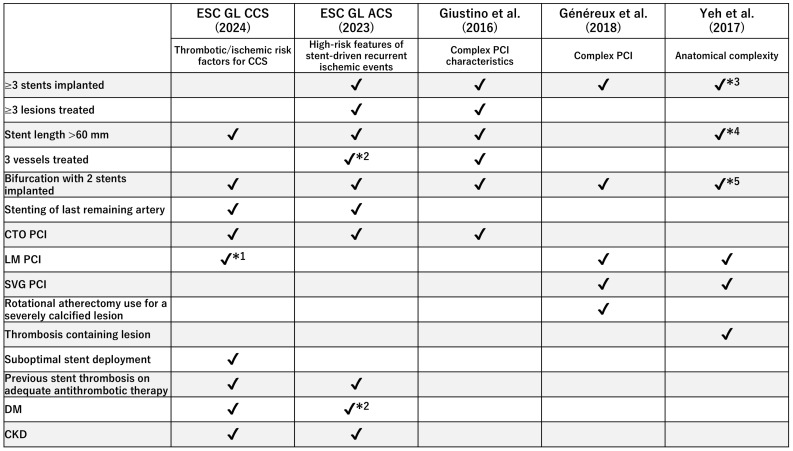
Criteria of complex PCI. *^1^ Including proximal LAD, *^2^ diffuse multivessel disease, especially in patients with DM, *^3^ >2 lesions per vessel, *^4^ lesion length ≥ 30 mm, and *^5^ bifurcation with side branch ≥ 2.5 mm. ACS = acute coronary syndrome, CCS = chronic coronary syndrome, CKD = chronic kidney disease, CTO = chronic total occlusion, DM = diabetes mellitus, ESC = European Society of Cardiology, GL = guidelines, LAD = left anterior descending artery, LM = left main, PCI = percutaneous coronary intervention, and SVG = saphenous vein graft [3,4,5,6,7].

Although “complex PCI” lacks a universally accepted definition, this concept has been consistently used across clinical trials and guidelines. Common features include treatment of ≥3 lesions, implantation of ≥3 stents, total stent length ≥ 60 mm, multivessel disease, and complex revascularization such as LM disease, CTO, saphenous vein graft, bifurcation stenting requiring two-stent techniques (e.g., double kissing crush, culotte, T-stenting), severe calcification requiring atherectomy (rotational, orbital, or intravascular lithotripsy), and last patent vessel (Figure 1). These criteria were defined on the basis of previously published reports linking features of procedural complexity with ischemic risk and by the expert opinions [3]. The thrombotic/ischemic risk factors, defined by the European Society of Cardiology (ESC) guidelines on chronic coronary syndrome (CCS) and acute coronary syndrome (ACS), also include patient-related factors such as prior stent thrombosis on adequate antiplatelet therapy, diabetes requiring medication, and chronic kidney disease, in addition to the aforementioned procedure-related factors [4,5]. Of note, the emerging concept of complex high-risk indicated PCI (CHIP PCI) reflects patient-specific characteristics (e.g., advanced age, frailty, severe left ventricular dysfunction, or chronic kidney disease) and procedure-specific characteristics (e.g., mechanical circulatory support) and is conceptually different from complex PCI.

Complex PCI is associated with several pathophysiologic mechanisms that may increase ischemic risk. Longer procedural time, more complex procedures, and more atherosclerotic burden, often involved in extensive coronary artery disease, increase the risk of early ischemic events. Higher likelihood of suboptimal stent results at the acute phase, such as underexpansion and malapposition due to lesion complexity (e.g., severe calcification, diffuse lesion, and bifurcation lesion) and incomplete revascularization, may contribute to both early and late ischemic risks such as stent thrombosis and restenosis. Increased metal burden and delayed endothelialization may delay vessel healing and increase the risk of late stent failure.

## 4. Risk of Bleeding in Patients Undergoing Complex PCI

In contemporary PCI practice, major bleeding is considered a key prognostic event. Previous large registries and randomized trials have consistently demonstrated that major bleeding was strongly associated with increased risk of all-cause and cardiovascular mortality [8,9,10]. Of note, the excess mortality risk following a bleeding event is comparable to, or even greater than that observed after MI [8]. Several mechanisms account for the profound prognostic impact of bleeding in patients undergoing PCI. Major bleeding can lead to hemodynamic compromise and subsequent end-organ hypoperfusion, and it often necessitates interruption or de-escalation of antithrombotic therapy [6,11,12], thereby increasing the risk of ischemic events. In addition, bleeding triggers inflammatory and prothrombotic responses that further exacerbate cardiovascular vulnerability, while transfusion introduces additional risks such as thrombosis and immunomodulatory effects [13]. Collectively, these pathways contribute to an increased risk of mortality after a bleeding event.

Patients undergoing complex PCI often have multiple comorbidities, such as older age, chronic kidney disease, diabetes mellitus, and history of stroke, which constitute major criteria for high bleeding risk (HBR) (e.g., ARC-HBR [14], PRECISE-DAPT score [15]), and, consequently, the presence of these comorbidities inherently elevates the bleeding risk. Previous studies have reported that 30–50% of patients undergoing complex PCI meet HBR criteria [1,9], underscoring the frequent overlap of ischemic and bleeding risks in this population. Due to these competing risks of ischemia and bleeding, patients undergoing complex PCI represent a population presenting a clinical dilemma with regard to tailoring the DAPT regimen.

Which risk should be prioritized when determining the DAPT regimen? A meta-analysis of eight randomized controlled trials (RCTs) that included 14,963 patients undergoing PCI demonstrated that, among non-HBR patients, long-term DAPT (12 or 24 months) reduced ischemic events in both complex (absolute risk difference: −3.86%, 95% confidence interval [CI]: −7.71 to +0.06) and non-complex PCI groups (absolute risk difference: −1.14%, 95% CI: −2.26 to +0.02) compared with short-term DAPT (3 or 6 months). In contrast, patients at HBR derived no ischemic or mortality benefit from long-term DAPT, irrespective of PCI complexity [16]. Collectively, these findings suggest that bleeding risk may play a more relevant role than ischemic risk in guiding decisions on DAPT duration. The current ESC guidelines recommend first assessing the bleeding risk, followed by consideration of ischemic risk when determining the DAPT duration [4,5]. From the perspective of patients undergoing complex PCI, these findings also suggest that maintaining DAPT for 12 months or longer may be advantageous, provided that they do not meet criteria for HBR.

## 5. Antithrombotic Therapy Strategies

### 5.1. Current Guideline Recommendation

**Figure 2:**
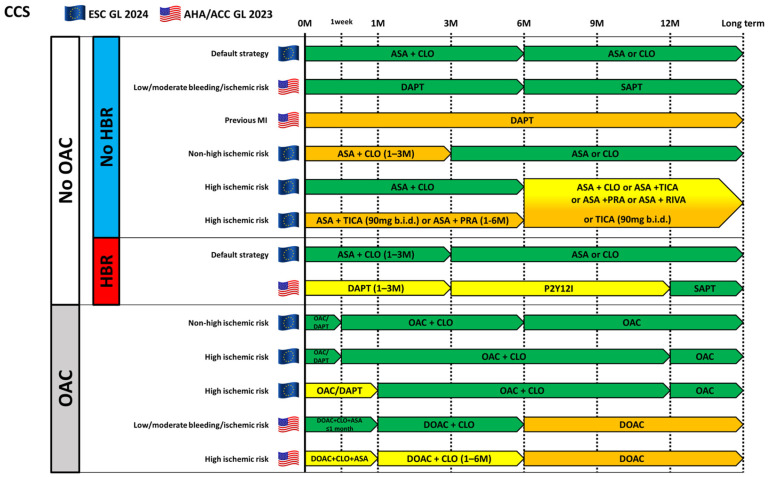
Overview of guideline recommendations for CCS patients. Colors correspond to the class of recommendations (green: Class I, yellow: Class IIa, orange: Class IIb). ACC = American College of Cardiology, AHA = American Heart Association, CCS = chronic coronary syndrome, DAPT = dual antiplatelet therapy, DOAC = direct oral anticoagulant, ESC = European Society of Cardiology, GL = guidelines, HBR = high bleeding risk, MI = myocardial infarction, OAC = oral anticoagulation, P2Y12I = P2Y12 inhibitor, and SAPT = single antiplatelet therapy [4,17].

**Figure 3:**
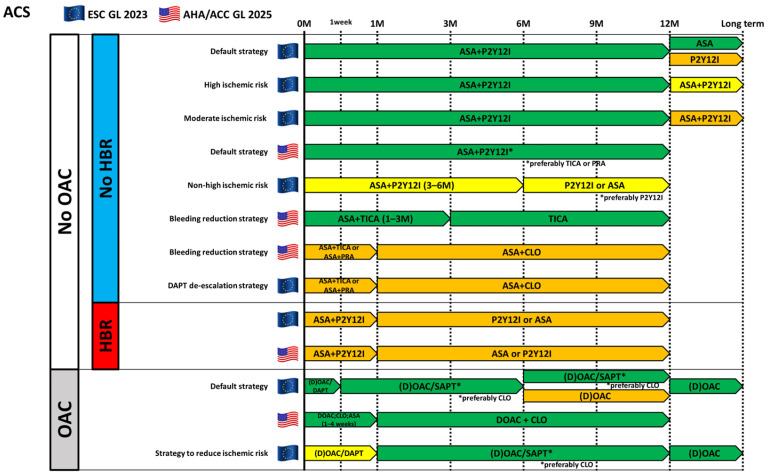
Overview of guideline recommendations for ACS patients. Colors correspond to the class of recommendations (green: Class I, yellow: Class IIa, orange: Class IIb). ACC = American College of Cardiology, ACS = acute coronary syndrome, AHA = American Heart Association, DAPT = dual antiplatelet therapy, DOAC = direct oral anticoagulant, ESC = European Society of Cardiology, GL = guidelines, HBR = high bleeding risk, OAC = oral anticoagulation, P2Y12I = P2Y12 inhibitor, and SAPT = single antiplatelet therapy [4,18].

In patients undergoing PCI, DAPT with aspirin and a P2Y12 inhibitor, administered for a variable duration tailored to individual risk profiles, remains the cornerstone of secondary prevention. The current guideline recommendations for DAPT regimens are summarized in Figure 2 and Figure 3. In high ischemic CCS patients without HBR or oral anticoagulation (OAC) indication, clopidogrel DAPT for 6 months (Class I) or ticagrelor/prasugrel DAPT for 1–6 months (Class IIb), followed by clopidogrel/ticagrelor/prasugrel DAPT (Class IIa), dual pathway inhibition (DPI) (i.e., aspirin plus rivaroxaban 2.5 mg b.i.d.) (Class IIa), or ticagrelor monotherapy (Class IIb), is recommended by the ESC guidelines (Figure 2) [4]. The American Heart Association (AHA)/American College of Cardiology (ACC) guidelines consider extended DAPT beyond 12 months for a period of up to 3 years as an option for CCS patients with a previous MI and at low bleeding risk to reduce major adverse cardiovascular events (MACE) (Class IIb) [17]. In high ischemic CCS patients with an OAC indication, continuation of aspirin up to 1 month after PCI in addition to OAC and clopidogrel (Class IIa) is recommended by both ESC and AHA/ACC guidelines (Figure 2). After up to one month of triple therapy, the ESC guidelines recommend 12 months of OAC plus clopidogrel, while the AHA/ACC guidelines recommend 6 months, with both guidelines subsequently endorsing OAC monotherapy. Following PCI in ACS patients, a default DAPT regimen consisting of a potent P2Y12 receptor inhibitor (prasugrel or ticagrelor) and aspirin is generally recommended for 12 months, irrespective of the stent type, unless there are contraindications (Figure 3) [5,18]. In the ESC guidelines, for extended long-term secondary prevention, adding a second antithrombotic agent to aspirin is recommended for patients with high ischemic risk and no HBR (Class IIa), whereas it may be considered for those with moderate ischemic risk in the absence of HBR (Class IIb). Collectively, prolonged DAPT is appropriate for patients who are not at HBR but exhibit high ischemic risk, such as those undergoing complex PCI. Importantly, in routine clinical practice, 20–40% of patients continue DAPT beyond 1 year post-PCI, and complex PCI was significantly associated with prolonged DAPT use [19].

### 5.2. P2Y12 Inhibitor Monotherapy

Published studies on antithrombotic therapies in patients undergoing complex PCI are summarized in Table 1. Different types of P2Y12 inhibitors and various timings for aspirin withdrawal after PCI have been evaluated in RCTs, consistently demonstrating an overall favorable balance of safety and efficacy. Recent meta-analyses of RCTs demonstrated that P2Y12 inhibitor monotherapy after a short course of DAPT (1–3 months) compared with standard DAPT showed a similar risk of cardiovascular events without interaction between complex and non-complex PCI [20,21]. The risk of major bleeding was significantly reduced by P2Y12 inhibitor monotherapy in both complex and non-complex PCI cohorts. According to the contemporary guidelines, ticagrelor monotherapy is recommended after 1–6 months of DAPT in high ischemic CCS patients without HBR (class IIb) [5] and after 1–3 months of ticagrelor DAPT in ACS patients without HBR (Class I) [18] (Figure 2 and Figure 3).

Drug-specific findings, however, are heterogeneous. Clopidogrel monotherapy has been evaluated in three RCTs [33,34,35]. In the SMART-CHOICE and STOPDAPT-2 trials, in which 40–60% of patients presented with ACS, clopidogrel monotherapy after 1 or 3 months of DAPT consistently reduced major bleeding without increasing ischemic events compared with standard 12-month DAPT. In contrast, the STOPDAPT-2 ACS trial demonstrated that clopidogrel monotherapy after 1 to 2 months of DAPT failed to demonstrate noninferiority to 12-month DAPT for the net clinical benefit due to a numerical increase in cardiovascular events (hazard ratio [HR] 1.50, 95%CI 0.99–2.26) despite a reduction in bleeding events (HR 0.46, 95%CI 0.23–0.94). In a pooled analysis of the STOPDAPT-2 total cohort comprising approximately 6000 patients, clopidogrel monotherapy following 1-month DAPT demonstrated a numerically higher incidence of cardiovascular events in patients with ACS, which was not observed in those with CCS, raising the possibility of a safety concern when applying clopidogrel monotherapy in the ACS population [36]. In addition, the timing of aspirin withdrawal (1–2 months vs. 3 months of DAPT) may also have contributed to the differing results between the SMART-CHOICE and STOPDAPT-2 ACS trials. In subgroup analyses of patients undergoing complex PCI, clopidogrel monotherapy following 1–3 months of DAPT did not result in a significant increase in ischemic events [22,23,37].

By contrast, multiple trials of ticagrelor monotherapy consistently showed reduced major bleeding with preserved ischemic safety, with effects generally consistent across strata of procedural complexity. In the GLOBAL LEADERS trial, ticagrelor monotherapy after 1-month DAPT was compared with 12-month DAPT using ticagrelor or clopidogrel followed by aspirin monotherapy among 15,968 patients undergoing PCI [38]. A post hoc subgroup analysis revealed a significant reduction in the composite primary endpoint of all-cause death or new Q-wave MI with ticagrelor monotherapy in the complex PCI subgroup, with no reduction in non-complex PCI patients (*p* for interaction = 0.015). The observed benefit was largely attributable to a significant interaction among ACS patients, with no significant difference in bleeding outcomes [24]. The T-PASS trial compared ticagrelor monotherapy after <1-month DAPT with 12-month ticagrelor DAPT among 2850 ACS patients undergoing PCI [39]. Ticagrelor monotherapy after <1-month DAPT was superior to 12-month DAPT for the 1-year composite outcome of death, MI, stent thrombosis, stroke, and major bleeding, primarily due to a significant reduction in major bleeding (2.8% vs. 5.2%, *p* = 0.002). No significant interaction was observed according to the presence of multivessel disease or total stent length. The ULTIMATE-DAPT trial similarly showed that ticagrelor monotherapy after 1-month DAPT reduced clinically relevant bleeding (2.1% vs. 4.6%, *p* < 0.001) while maintaining comparable rates of MACE (3.6% vs. 3.7%, *p* = 0.89) compared with ticagrelor DAPT for 12 months among 3400 ACS patients undergoing PCI, with no evidence of interaction by PCI complexity (i.e., multivessel disease or the total stent length). Ticagrelor monotherapy after 3-month DAPT was evaluated in 7119 high-risk patients enrolled in the TWILIGHT trial [40] and in 3056 ACS patients in the TICO trial [41]. In the TWILIGHT-COMPLEX post hoc substudy, which included 2342 patients undergoing complex PCI, ticagrelor monotherapy, consistent with the findings of the main trial, significantly reduced major bleeding while maintaining a comparable ischemic risk relative to ticagrelor DAPT, irrespective of procedural complexity [25]. The post hoc subgroup analysis of the TICO trial showed no significant interaction according to PCI complexity, which incorporated both clinical characteristics such as diabetes and chronic kidney disease and procedural complexity criteria [26]. In a pooled analysis of 7529 ACS patients from the TWILIGHT and TICO trials, ticagrelor monotherapy significantly reduced major bleeding, without increasing adverse clinical events; importantly, these effects were consistent across strata of PCI complexity, with no evidence of interaction [42].

Low-dose prasugrel monotherapy (maintenance 3.75 mg/day), a Japan-specific reduced dose intended to preserve antiplatelet efficacy while mitigating bleeding risk, was evaluated in 6002 patients with ACS or HBR in the STOPDAPT-3 trial [43]. At 1 month after PCI, the prasugrel monotherapy group was not superior to the DAPT group for major bleeding (4.5% vs. 4.7%, *p* = 0.66). Although prasugrel monotherapy was non-inferior to the DAPT group for cardiovascular events (4.1% vs. 3.7%, *p* for noninferiority = 0.01), there was an increased risk of unplanned revascularization and subacute stent thrombosis, potentially reflecting insufficient platelet inhibition with low-dose prasugrel compared with more potent P2Y12 inhibitors, such as ticagrelor or the standard dose of prasugrel. In a prespecified subgroup analysis that included 1230 patients with ACS or HBR undergoing complex PCI, low-dose prasugrel monotherapy demonstrated similar ischemic and bleeding outcomes at 1 month relative to DAPT [27].

In a post hoc analysis of the HOST-IDEA trial, which evaluated the efficacy and safety of 3 to 6 months of DAPT (mainly clopidogrel) compared with 12-month DAPT among 1992 patients treated with third-generation DES, shortened DAPT significantly reduced major bleeding in the complex PCI subgroup (*p* for interaction = 0.029) without increasing ischemic risk [28].

Collectively, P2Y12 inhibitor monotherapy has been shown not to increase ischemic events in patients undergoing complex PCI, which is in line with the findings in non-complex PCI, while consistently reducing bleeding risk. In the overall study cohort, ticagrelor monotherapy reduced bleeding events without increasing ischemic events, whereas clopidogrel monotherapy has shown signals of excess ischemic risk in ACS populations, particularly when aspirin is discontinued within 1–2 months. In contrast, an aspirin-free strategy using low-dose prasugrel did not demonstrate an increase in ischemic events but failed to achieve a significant reduction in bleeding. Although physicians should be aware that most available data on patients undergoing complex PCI are derived from post hoc subgroup analyses of RCTs, as supported in current guideline recommendations, P2Y12 inhibitor monotherapy represents an important therapeutic option, even in patients treated with complex PCI.

For long-term secondary prevention after PCI, although lifelong aspirin therapy has been recommended when not contraindicated, P2Y12 inhibitor monotherapy is increasingly recognized as a novel alternative antiplatelet strategy. In a patient-level meta-analysis synthesizing seven RCTs of patients with established coronary artery disease, P2Y12 inhibitor monotherapy for long-term secondary prevention demonstrated more favorable ischemic outcomes than aspirin monotherapy, without increasing major bleeding [44]. Consistent with these findings, both the HOST-EXAM trial and the SMART-CHOICE 3 trials showed that clopidogrel monotherapy reduced thrombotic events without increasing bleeding, and importantly, neither trial demonstrated a significant interaction according to PCI complexity [45,46]. However, in the secondary analysis of STOPDAPT-3, conducted exclusively in 5833 ACS patients, clopidogrel monotherapy after 1-month DAPT did not demonstrate superiority over aspirin monotherapy (cardiovascular events: 4.5% vs. 4.5%, *p* = 0.97, major bleeding: 2.0% vs. 1.9%, *p* = 0.92), and subgroup analysis suggested a possible advantage of aspirin in the complex PCI group [47]. These discrepancies likely reflect differences in clinical presentation, timing of transition to monotherapy, follow-up duration, and endpoint definitions. While the overall direction of evidence supports P2Y12 inhibitor monotherapy (particularly clopidogrel), the data for complex PCI are derived from post hoc subgroup analyses, and thus the strength of evidence in complex PCI remains limited.

### 5.3. Prolonged Antiplatelet Therapy

The DAPT trial evaluated 18 additional months of DAPT versus aspirin alone among patients who had no adverse events during the first year following PCI. In a post hoc subgroup analysis of 3730 patients undergoing complex PCI, extended DAPT was significantly associated with a lower incidence of the composite endpoint of death, MI, or stroke (4.7% vs. 6.3%; HR 0.72, 95% CI 0.55–0.96), with no significant excess in GUSTO moderate/severe bleeding (2.2% vs. 1.6%; HR 1.41, 95% CI 0.87–2.28) [7]. No significant interaction by PCI complexity was observed for either ischemic or bleeding events. The current guidelines recommend prolonged antiplatelet therapy only for patients without HBR and with high ischemic risk.

### 5.4. De-Escalation and Escalation of Antithrombotic Therapies

Escalation and de-escalation strategies have been evaluated using both guided (CYP2C19 genotyping or platelet function testing) and unguided approaches. Across six RCTs (two guided by platelet function testing, one guided by genotyping, and three unguided), a de-escalation approach has been assessed in patients presenting with ACS [48]. With the exception of one study [49], each trial achieved its primary endpoint and demonstrated significant reductions in net adverse clinical events and major bleeding without an increase in ischemic complications. A recent individual patient-level meta-analysis pooling four of these trials and including 10,133 patients confirmed that both guided and unguided de-escalation yielded consistent benefits, lowering both ischemic and bleeding outcomes [50]. No interaction was observed for either endpoint according to procedural complexity (multivessel- versus single-vessel PCI or implantation of ≥3 versus <3 stents). Similarly, no heterogeneity by PCI complexity was observed in the HOST-REDUCE-POLYTECH [29] and TALOS-AMI [30] trials, which showed reduced bleeding with unguided prasugrel or ticagrelor de-escalation. Given these findings, the current ESC ACS guidelines endorse de-escalation after the first 30 days to reduce bleeding risk (Class IIb, Level A) (Figure 3) [5].

By contrast, evidence for the escalation strategy is more heterogeneous. A meta-analysis of both randomized and non-randomized trials showed that the guided escalation was associated with a reduced risk of ischemic events without a corresponding increase in bleeding, but these findings are limited by study design heterogeneity and inclusion of non-randomized data [51]. Recently, the TAILORED-CHIP study investigated a tailored antiplatelet therapy with early ticagrelor escalation (low-dose ticagrelor at 60 mg twice daily plus aspirin < 6 months) and late clopidogrel de-escalation (clopidogrel monotherapy > 6 months) in 2018 patients with high-risk anatomical or clinical characteristics undergoing complex PCI [31]. The tailored strategy did not reduce the 12-month composite of ischemic and bleeding events compared with standard 12-month DAPT (10.5% vs. 8.8%, *p* = 0.21). Major ischemic outcomes were similar between groups, while bleeding tended to be higher with the tailored approach (7.2% vs. 4.8%).

Clinically, de-escalation appears a reasonable approach to lower bleeding in many patients after 30 days, whereas escalation should be considered selectively in patients at high ischemic risk and preferably guided by testing [52]. Adequately powered prospective trials focused on complex PCI are needed to define optimal escalation and de-escalation algorithms in this high-risk population.

### 5.5. Dual Pathway Inhibition

DPI refers to the combined use of low-dose anticoagulation and antiplatelet therapy to simultaneously inhibit thrombin generation and platelet activation, offering an intensified antithrombotic effect to prevent ischemic events. This strategy has been proposed as an alternative approach for post-PCI pharmacologic management.

The APPRAISE-2 trial using standard-dose direct oral anticoagulant (DOAC) added to DAPT demonstrated excess major bleeding without ischemic benefit compared with DAPT alone in 7392 patients with ACS who exhibited high-risk clinical features (44% of whom underwent PCI) [53]. The GEMINI-ACS-1 trial subsequently examined the regimen of low-dose rivaroxaban (i.e., 2.5 mg twice daily) added to a P2Y12 inhibitor compared with standard DAPT in 3037 ACS patients. Rates of clinically significant bleeding at 12 months were comparable between the two groups (5% vs. 5%, *p* = 0.58); however, the trial was underpowered to assess ischemic outcomes [54]. The COMPASS trial evaluated low-dose rivaroxaban plus aspirin versus aspirin alone in patients with stable atherosclerotic cardiovascular disease and showed that DPI reduced major cardiovascular events and mortality at the cost of increased major bleeding [55]. A prespecified subgroup analysis of 9862 patients with prior PCI (38% multivessel PCI) showed that DPI lowered the incidence of MACE (4.0% vs. 5.5%; HR 0.74, 95% CI 0.61–0.88) and all-cause mortality (2.5% vs. 3.5%; HR 0.73, 95% CI 0.58–0.92), while increasing major bleeding (3.3% vs. 2.0%; HR 1.72, 95% CI 1.34–2.21) [56]. Treatment effects were consistent regardless of whether patients had undergone single- or multivessel PCI (*p* for interaction = 0.31). Interestingly, adding low-dose rivaroxaban reduced stroke risk (an effect not observed with prolonged DAPT) although no significant reduction in MI was observed. Collectively, DPI should be considered selectively in non-HBR patients with high ischemic risk, and further prospective studies are needed to define the role of DPI specifically after complex PCI.

### 5.6. Patients Requiring Oral Anticoagulation

Approximately 20% of patients with atrial fibrillation (AF) undergo PCI and therefore require OAC for stroke prevention in addition to DAPT to mitigate the risks of stent thrombosis and MI [57]. The triple antithrombotic therapy (i.e., a combination of an OAC plus DAPT) substantially increases bleeding risk and is a major criterion for HBR [14]. Across five RCTs, double antithrombotic therapy, compared with triple therapy, significantly reduced major or clinically relevant non-major bleeding without increasing ischemic events, supporting the recommendation of double therapy (OAC plus a P2Y12 inhibitor, predominantly clopidogrel) after 1–4 weeks of triple therapy in patients with AF undergoing PCI [58,59,60,61,62]. A post hoc analysis of PIONEER AF-PCI and RE-DUAL PCI trials demonstrated that the relative treatment effects of DAPT and/or DOAC-based regimens versus vitamin K antagonist-based triple therapy were consistent across the spectrum of PCI complexity [63,64].

However, ischemic safety remains a concern. Although individual studies were underpowered to detect differences in ischemic events, two meta-analyses showed that dual antithrombotic therapy significantly increased the risk of stent thrombosis [65,66]. Accordingly, the current guidelines recommend the continuation of aspirin for up to 1 month after PCI, in addition to OAC and clopidogrel in patients with high ischemic risk or with anatomical/procedural features judged to outweigh bleeding concerns (Class IIa) (Figure 2 and Figure 3) [4,5,17]. Taken together, the evidence supports routine early transition to double therapy to reduce bleeding in most AF patients after PCI; however, clinicians should individualize treatment for those with high ischemic risk or complex anatomy because of the trade-off between bleeding and stent thrombosis.

## 6. Specific Patient/Lesion Subsets

### 6.1. Bifurcation PCI

Patients with coronary bifurcation lesions represent 15–20% of those undergoing PCI and carry an increased risk of both periprocedural and long-term adverse events [67]. Definite or probable stent thrombosis occurs predominantly within the first 30 days, with an overall incidence of 1.5–2%, which is nearly twice that observed after non-bifurcation PCI (<1%) [67]. The risk of stent thrombosis increases with lesion complexity, and the use of a two-stent technique confers approximately twice the stent thrombosis risk compared with a single-stent strategy [68]. Indeed, bifurcation PCI with the two-stent technique has consistently been incorporated into most definitions of complex PCI. Among the 2082 patients in the COBIS II registry who underwent bifurcation stenting (two-stenting: 26% of patients) and remained without clinical events at 12 months, prolonged DAPT resulted in a reduced 4-year incidence of all-cause death, MI, and stent thrombosis compared with short-term DAPT (<12 months) [69]. This benefit of extended therapy appeared consistent regardless of the lesion location or stenting technique. Other observational studies investigating the impact of DAPT duration on ischemic outcomes among patients undergoing bifurcation PCI (two-stenting: 10–20% of patients) consistently demonstrated that prolonged DAPT was associated with reduced ischemic outcomes compared with a shorter DAPT duration [70,71,72]. A meta-analysis of six observational studies that included 12,000 patients who underwent PCI for coronary bifurcation lesions showed that prolonged DAPT (>6 months) significantly reduced the risks of MACE, death, and stent thrombosis compared with short-term DAPT (≤6 months) [32]. However, because these findings are derived from observational studies rather than RCTs, caution is warranted in their interpretation. In the absence of definitive RCT data, it remains difficult to draw definitive conclusions for this specific patient subset, and DAPT regimens should be individualized with careful consideration given to bleeding risk. Nonetheless, given the inherently higher risk of stent thrombosis associated with bifurcation two-stent techniques, a longer duration of DAPT is generally considered reasonable until more robust evidence becomes available.

### 6.2. LM PCI

As the LM coronary artery supplies a large proportion of the left ventricular myocardium, stent failure in this segment carries a high likelihood of unfavorable outcomes. Accordingly, LM PCI is considered one of the highest-risk subsets within contemporary PCI practice. In the IDEAL-LM study that included 818 patients undergoing LM PCI, a biodegradable-polymer everolimus-eluting stent followed by 4-month DAPT demonstrated noninferiority compared with a durable-polymer everolimus-eluting stent followed by 12 months of DAPT, although a numerical trend toward higher ischemic event rates was observed in the biodegradable polymer stent with the 4-month DAPT group [73]. Propensity score-adjusted analyses from the EXCEL trial indicated that an extension of DAPT beyond 1 year did not provide additional clinical benefit, whereas a signal toward harm emerged with extended DAPT [74]. In a post hoc subgroup analysis of the PRODIGY trial that included 953 patients with LM or proximal left anterior descending artery disease, 24-month DAPT did not reduce the composite outcome of death, MI, or stroke compared with 6-month DAPT, although it was associated with a lower incidence of definite, probable, or possible stent thrombosis (2.8% vs. 5.6%, *p* = 0.02) [75]. In PCI for LM, it also remains difficult to draw definitive conclusions regarding the optimal antiplatelet regimen due to the limited evidence. When optimal results can be achieved with simple stenting of the LM shaft lesion, applying antiplatelet strategies typically recommended for complex PCI may not be necessary. However, once LM bifurcation stenting is required, procedural complexity clearly increases and the risk of critical stent thrombosis may substantially increase, and thus a longer duration of DAPT may be reasonable.

### 6.3. CTO PCI

CTOs are frequently characterized by long lesion length and marked calcification, factors that require longer stent implantation and increase the risk of stent underexpansion, which predisposes to stent failure. In addition, CTO-related vessel shrinkage and subsequent chronic lumen enlargement can lead to implantation of undersized stents, resulting in late acquired malapposition and thereby increasing the risk of late stent thrombosis. In such high-risk settings, extending the duration of DAPT would be even more reasonable. In this regard, CTO is listed as one of the “thrombotic/ischemic risk factors for CCS” and as an “anatomical/procedural thrombotic risk characteristic” defined in the ESC guidelines [4]. Previous observational studies that include patients undergoing PCI for CTO lesions have demonstrated a decreased risk of ischemic events by long-term DAPT compared with short-term DAPT [76,77]. Further studies are warranted to define the optimal antithrombotic strategy in this patient subset.

## 7. Summary and Future Perspective

Complex PCI has been increasingly performed due to advances in PCI technology and expanding indications and is now performed frequently in routine clinical practice. Although complex PCI is strongly linked with an increased risk of ischemic events, the current guidelines recommend that bleeding risk should be prioritized over PCI complexity when deciding on the antiplatelet regimen. In patients undergoing complex PCI without bleeding risk, prolonged DAPT is considered a reasonable treatment option, whereas potent P2Y12 inhibitor monotherapy (i.e., ticagrelor) or DPI are potential alternatives. In most antithrombotic therapy trials, subgroup analyses have not demonstrated any significant interaction according to PCI complexity.

Caution is warranted when interpreting the evidence summarized in this review because much of the evidence in complex PCI populations was derived from post hoc subgroup analyses that may lack sufficient power to support lesion- or complexity-specific conclusions. In addition, the generalizability of the study findings should be interpreted carefully. Several included studies were conducted mainly in East Asian populations, used reduced-dose prasugrel, or enrolled selected non-HBR patients. These characteristics may limit the applicability of some results to broader international practice, underscoring the need for further studies in more diverse populations.

While the criteria for “complex PCI” largely reflect the underlying severity of coronary artery disease, components such as total stent length, number of stents, and number of treated lesions are procedural outcomes and therefore are at least partly modifiable. This underscores the clinical importance of using a physiological assessment to avoid unnecessary complex PCI that may undesirably increase a patient’s ischemic risk [78]. When complex PCI is clinically required, intravascular imaging needs to be employed to optimize the procedural results and thereby minimize the subsequent ischemic risk. Indeed, a recent meta-analysis showed that, among patients undergoing complex PCI, the use of intracoronary imaging significantly reduced ischemic events, especially stent thrombosis and repeat revascularization, compared with angiography alone [79]. Optimized PCI results may influence the acceptable duration and intensity of antithrombotic therapy, especially in HBR patients. Optimal PCI results may allow for the safe shortening of DAPT duration and a reduction in its intensity.

**Figure 4:**
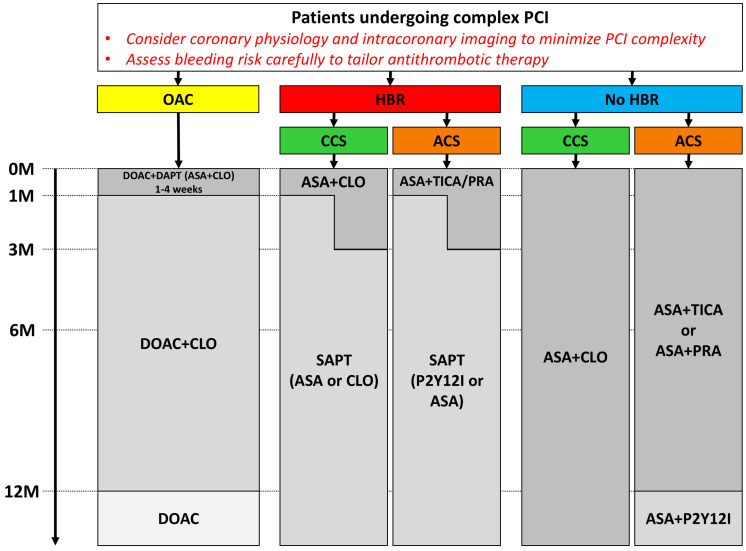
Proposed algorithm for antithrombotic therapy in patients undergoing complex PCI. ACS = acute coronary syndrome, CCS = chronic coronary syndrome, DAPT = dual antiplatelet therapy, HBR = high bleeding risk, OAC = oral anticoagulation, DOAC = direct oral anticoagulant, PCI = percutaneous coronary intervention, P2Y12I = P2Y12 inhibitor, and SAPT = single antiplatelet therapy.

A simplified proposed algorithm is presented to guide decision-making for antithrombotic therapy after complex PCI (Figure 4). In patients scheduled for complex PCI, the use of coronary physiology and intracoronary imaging should be encouraged to minimize procedural complexity, particularly in those with HBR. In patients treated with OAC, up to one month of triple therapy followed by one year of dual therapy (DOAC and clopidogrel) should be regarded as the standard strategy. In HBR patients, single antiplatelet therapy after 1–3 months of DAPT is likely to represent the current standard of care. In patients without HBR, prolonged DAPT remains the gold-standard antithrombotic strategy.

Currently, several RCTs of antithrombotic therapy in patients undergoing complex PCI are ongoing. The E5TION trial (NCT04734353) is evaluating a tailored antiplatelet strategy comparing low-dose prasugrel (5 mg daily) with low-dose ticagrelor (60 mg twice daily) in East Asian patients undergoing CHIP PCI. The SMART-ATTEMPT trial (NCT04014803) is a prospective, open-label, multicenter randomized study designed to compare the efficacy and safety of aspirin plus prasugrel versus aspirin plus clopidogrel in patients undergoing complex PCI. These ongoing studies are expected to provide clinically relevant evidence on tailored antithrombotic strategies, thereby facilitating more personalized treatment options specifically for patients undergoing high-risk complex PCI. In addition, given the absence of a universally standardized definition of complex PCI, further validation and refinement of the criteria are warranted. As novel PCI devices continue to emerge and risk-prediction tools, including refined ischemic and bleeding scores and AI-driven algorithms, advance, antithrombotic therapy will increasingly shift toward precision medicine tailored to each patient’s unique risk profile. Accordingly, ongoing research in antithrombotic strategies remains essential to optimize outcomes in this high-risk population.

## Figures and Tables

**Table 1:** Overview of published studies on antithrombotic therapies in patients undergoing complex PCI.

Study Name/Study Design	Year	n (%) of ACS	n (%) of Complex PCI	Complex PCI Criteria	Follow-Up Period	Ischemic/Bleeding Endpoints	Treatment	Ischemic Event Rate in Complex PCI Group		*p*_for interaction_ (Complex vs. Non-complex)	Bleeding Event Rate in Complex PCI Group		*p*_for interaction_ (Complex vs. Non-Complex)
P2Y12 inhibitor monotherapy													
SMART-CHOICE [22]: Post hoc subgroup analysis	2021	2993 (100%)	498 (16.6%)	3 vessels treated ≥3 stents implanted ≥3 lesions treated Bifurcation with 2 stents implanted Total stent length > 60 mm	12 months	Death, MI, or stroke; BARC 2, 3, or 5 bleeding	P2Y12I monotherapy after 3-month DAPT	3.8%	*p* = 0.853	0.483	1.9%	*p* = 0.340	0.904
							12-month DAPT	4.2%			3.4%		
STOPDAPT-2 [23]: Prespecified subgroup analysis	2023	4136 (68.9%)	999 (16.7%)	3 vessels treated ≥3 stents implanted ≥3 lesions treated Bifurcation with 2 stents implanted Total stent length > 60 mm CTO PCI	12 months	cardiovascular death, MI, stroke, or stent thrombosis; TIMI major or minor bleeding	Clopidogrel monotherapy after 1-month DAPT	2.5%	*p* = 0.98	0.53	0.6%	*p* = 0.12	0.9
							12-month clopidogrel DAPT	2.5%			1.8%		
GLOBAL LEADERS [24]: Post hoc subgroup analysis	2019	7260 (45.4%)	4570 (28.6%)	3 vessels treated ≥3 stents implanted ≥3 lesions treated Bifurcation with 2 stents implanted Total stent length > 60 mm	24 months	Death or MI; BARC 3 or 5 bleeding	Ticagrelor monotherapy after 1-month DAPT	3.5%	*p* = 0.002	0.015	2.5%	*p* = 0.856	0.834
							Aspirin monotherapy after 12-month DAPT	5.4%			2.5%		
TWILIGHT [25]: Post hoc subgroup analysis	2020	4614 (64.8%)	2342 (32.8%)	3 vessels treated ≥3 stents implanted ≥3 lesions treated Bifurcation with 2 stents implanted Total stent length > 60 mm CTO PCI Atherectomy device use LM PCI Surgical bypass graft PCI	15 months	Death, MI, or stroke; BARC 2, 3, or 5 bleeding	Ticagrelor monotherapy after 3-month DAPT	3.8%	*p* = NA	0.13	4.2%	*p* = NA	0.79
							15-month ticagrelor DAPT	4.9%			7.7%		
TICO [26]: Post hoc subgroup analysis	2021	3056 (100%)	409 (13.3%)	≥3 stents implanted Total stent length > 60 mm Bifurcation with 2 stents implanted CTO PCI LM PCI DM or CKD	12 months	Death, MI, stroke, stent thrombosis, or target vessel revascularization; TIMI major bleeding	Ticagrelor monotherapy after 3-month DAPT	3.3%	*p* = NA	0.718	2.8%	*p* = NA	0.092
							12-month ticagrelor DAPT	4.5%			3.7%		
STOPDAPT-3 [27]: Prespecified subgroup analysis	2024	4474 (75%)	1228 (21%)	3 vessels treated ≥3 stents implanted ≥3 lesions treated Bifurcation with 2 stents implanted Total stent length > 60 mm CTO PCI	1 month	Cardiovascular death, MI, stroke, or stent thrombosis; BARC 3 or 5 bleeding	Low-dose prasugrel monotherapy	5.8%	*p* = NA	0.48	5.3%	*p* = NA	0.08
							1-month low-dose prasugrel DAPT	5.9%			3.7%		
HOST-IDEA [28]: Post hoc subgroup analysis	2025	1096 (55%)	624 (31%)	≥3 stents implanted ≥3 lesions treated Bifurcation with 2 stents implanted Total stent length > 60 mm LM PCI Heavy calcification	12 months	Cardiac death, target vessel MI, or clinically driven target lesion revascularization; BARC 3 or 5 bleeding	3 to 6-month DAPT	4.5%	*p* = 0.578	0.451	1.0%	*p* = 0.044	0.029
							12-month DAPT	5.5%			3.6%		
Prolonged antiplatelet therapy													
DAPT [7]: Post hoc subgroup analysis	2017	5359 (46.3%)	3730 (32.2%)	Unprotected LM ≥2 lesions per vessel Lesion length ≥ 30 mm Bifurcation lesion with Side branch ≥ 2.5 mm Saphenous vein graft Thrombotic lesions	30 months	MI or stent thrombosis; GUSTO moderate or severe bleeding	30-month DAPT	2.5%	*p* = 0.001	0.81	2.2%	*p* = 0.41	0.44
							12-month DAPT	4.5%			1.6%		
De-escalation and escalation of antithrombotic therapies													
HOST REDUCE POLYTECH [29]: Post hoc subgroup analysis	2022	2271 (100%)	705 (31.0%)	≥3 stents implanted ≥3 lesions treated Bifurcation PCI Total stent length > 60 mm LM PCI Heavy calcification	12 months	Cardiovascular death, MI, stent thrombosis, and repeat revascularization; BARC 2, 3, or 5 bleeding	DAPT with low-dose prasugrel (5 mg/day) after 1-month DAPT	5.3%	*p* = 0.70	0.84	1.8%	*p* = 0.002	0.08
							12-month DAPT with conventional prasugrel dose (10 mg/day)	6.0%			6.9%		
TALOS-AMI [30]: Post hoc subgroup analysis	2024	2697 (100%)	788 (29.2%)	Multivessel PCI ≥3 stents implanted ≥3 lesions treated Bifurcation with 2 stents implanted Total stent length >60 mm LM PCI DM or CKD	12 months	Cardiovascular death, MI, stroke, stent thrombosis, or ischemia-driven revascularization; BARC 2, 3, or 5 bleeding	Clopidogrel DAPT after 1-month ticagrelor DAPT	4.7%	*p* = 0.62	0.47	3.2%	*p* = 0.11	0.32
							12-month ticagrelor DAPT	5.3%			4.9%		
TAILORED-CHIP [31]: Primary analysis	2025	440 (21.8%)	2010 (99.9%)	LM PCI Bifurcation with 2 stents implanted CTO PCI Severe calcified lesions Lesion length ≥ 30 mm Multivessel PCI ≥3 stents implanted ≥3 lesions treated Total stent length > 60 mm	12 months	Death, MI, stroke, stent thrombosis, or target vessel revascularization; BARC 2, 3, or 5 bleeding	Clopidogrel DAPT after <6-month low-dose ticagrelor (60 mg twice daily) DAPT	3.9%	*p* = 0.25	NA	7.2%	*p* = 0.002	NA
							12-month DAPT	5.0%			4.8%		
LM PCI													
IDEAL-LM [32]: Primary analysis	2022	331 (40.4%)	818 (100%)	LM PCI	24 months	Death, MI, or target vessel revascularization; BARC 3 or 5 bleeding	4-month DAPT	14.6%	*p* = 0.1734	NA	2.7%	*p* = 0.0215	NA
							12-month DAPT	11.4%			0.5%		

ACS = acute coronary syndrome, CKD = chronic kidney disease, CTO = chronic total occlusion, DAPT = dual antiplatelet therapy, DM = diabetes mellitus, LM = left main, MI = myocardial infarction, NA = not available, PCI = percutaneous coronary intervention, and P2Y12I = P2Y12 inhibitor.

## Data Availability

No new data were created or analyzed in this study. Data sharing is not applicable to this article.
